# Health status of rescued people by the NGO Open Arms in response to the refugee crisis in the Mediterranean Sea

**DOI:** 10.1186/s13031-020-00275-z

**Published:** 2020-05-01

**Authors:** Guillermo Cañardo, Jesús Gálvez, Juanfe Jiménez, Núria Serre, Israel Molina, Cristina Bocanegra

**Affiliations:** 1 Open Arms NGO, Barcelona, Spain; 2 Tropical Medicine and International Health Unit Vall d’Hebron-Drassanes, Barcelona, PROSICS Barcelona Spain; 3 Infectious Diseases Department, University Hospital Vall d’Hebron, Barcelona, PROSICS Barcelona Spain

## Abstract

**Background:**

The migration over the Mediterranean has become one of the deadliest sea voyages in last few years. The NGO Open Arms works in the area since 2015, with the objectives of protecting and reporting human rights at sea. This paper aims to give an overview on characteristics and health conditions of rescued people by the NGO in the Central Mediterranean.

**Methods:**

A descriptive retrospective population study was conducted, including people who were rescued from distress at sea by the NGO Open Arms from 1st July 2016 to 31st December 2018.

**Results:**

In this period of time 22,234 people were rescued from sea. Among them 2234 (22.7%) were minors, and 177 (0.8%) pregnant women. The most frequent countries of origin were Nigeria (1278–13.1%), Eritrea (1215–12.3%) and Bangladesh (981–9.9%). Among all people rescued, 4516 (20.3%) reported symptoms. Scabies was the most frequent pathology, being suspected in 1817 (8.2%) people. Other infectious diseases were diagnosed in 91 (0.4%). Thirty-five (0.16%) patients suffered some complication from their chronic diseases. Acute injuries due to trauma, burns, aggressions, and bullet or bladed weapon wound were reported in 135 (0.6%) cases. Seventy-four corpses were recovered.

**Conclusions:**

Main diagnoses on board were directly related to the precarious living conditions through migratory route, violence and complications of chronic diseases due to lack of care. The large number of people rescued highlights the catastrophic effect on migrants’ health of European policies, which overlap the desire to restrict migratory movements on the humanitarian and health issues. An integrated information system and a coordinated response are basic to improve the situation in the area.

## Background

The migration over the Mediterranean has become one of the deadliest sea voyages in last few years. Since the beginning of 2015 the preferred route into Europe was through Turkey by road and onwards by boat to the Greek islands in the Turkish archipelago [1]. In 18th March 2016 a treaty between the European Union and Turkey was held [2], limiting the number of people using this route; since then, the route through Libya to Italy and Malta has been of greatest importance [3]. This route has increased substantially the serious risks immigrants take during their journeys. Reports from the UN refugee agency (UCHNUR) describe immigrants having survived the deadly desert crossing from Niger, kidnappings, torture, sexual abuse and detention in Libya [4], and the dangerous sea journey, in which 2.830 people are estimated to have died from January to June 2017 [5]; nevertheless the total number of deaths is not known, as many people is supposed to have drown with no witnesses.

In 2018, following the agreements signed between the European Union and Libya, and the blockade of the rescue efforts, the migratory route to reach Europe changed again, increasing the number of arrivals to Greece and Spain, and decreasing in the Central Mediterranean route to Italy. However, the danger of the crossings increased; it is estimated that in 2018, 2275 people died in the Mediterranean. Although the total number of deaths in the crossing decreased, the death rate by number of arrivals increased considerably, going from 1 in 38 in 2017 to 1 in 14 in 2018 [6].

The NGO Open Arms started its work in Lesbos Island, in Greece, in September 2015, as a consequence of this situation and the absence of response of the European Union. It is an NGO financed in more than 90% by private donations, made up of contracted professionals and volunteers in all areas necessary for this project. Its work consists mainly in the rescue of people in danger at sea; it also carries out tasks of information and awareness of the migratory reality in the Mediterranean. A fundamental part of their work is the report and protection of human rights at sea. In June 2016, and due to the shift in the main migratory route through the Mediterranean sea, it started to work in the Central Mediterranean with the boat Astral. In December 2016 a second ship, the Golfo Azzurro, joined the intervention, and in July 2017 a third vessel acquired by the NGO started to work as well in the Search and Rescue (SAR) Area. In 2018, a process of criminalization of rescue operations by certain European governments began, hardening the interventions and sometimes preventing landings in European ports. From the beginning of 2019, no rescue operation has been authorized by the European authorities, and since then, advocacy is been held to be able to come back to the SAR Area as soon as possible.

**Fig. 1:**
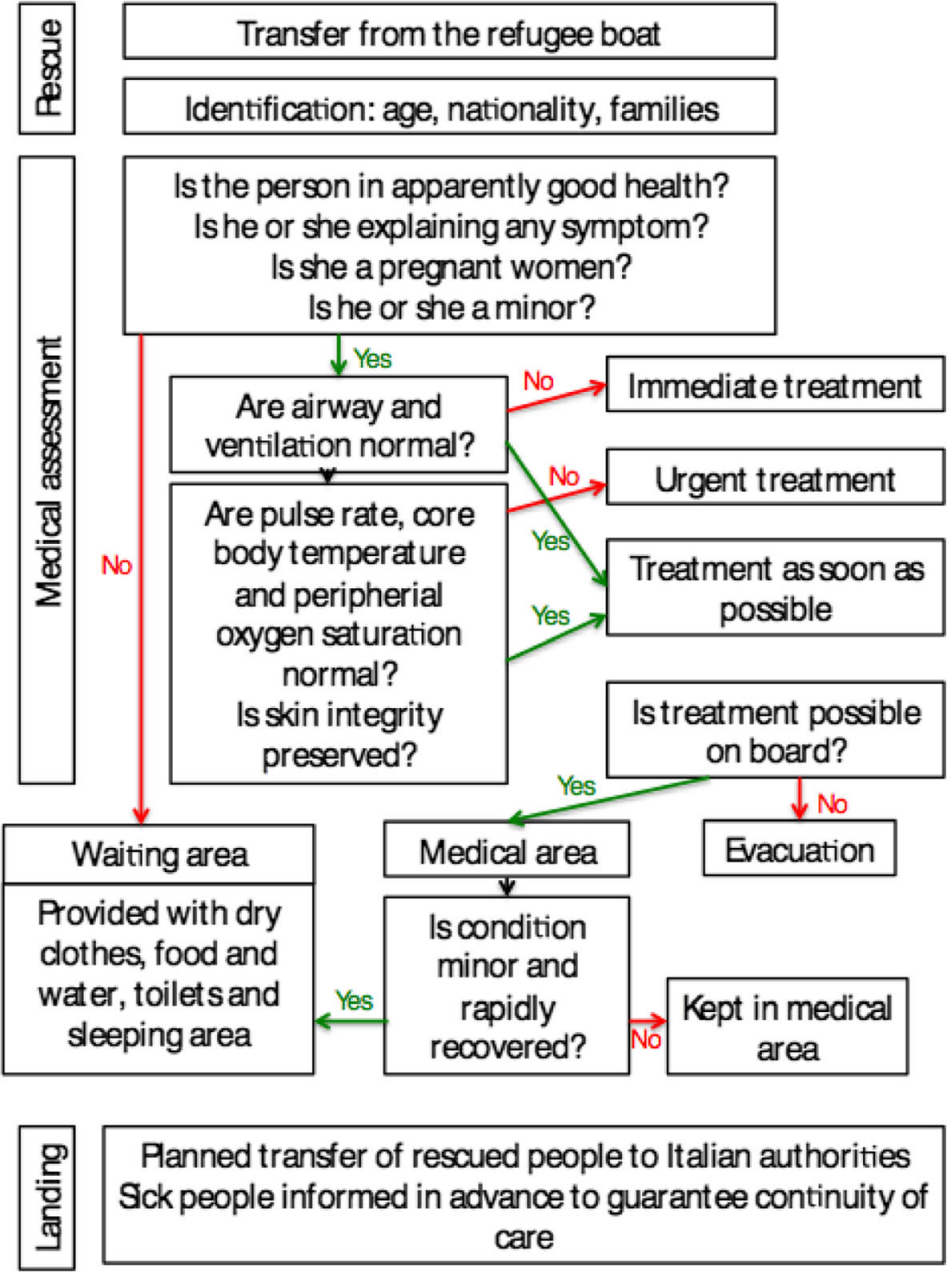
Chart of medical care on board. This flow is general; small changes could be made depending on the vessel capacity

All the NGO boats have a complete hospital on board; the medical service on board includes a team consisting in a doctor and a nurse and it was planned with a view to provide immediate medical assistance to a large number of people; handling and storage of corpses and assessment of causes of death also were taken into account as part of the medical field of work. The hospital has the capacity to handle all types of emergencies, including oral and parenteral treatments, probes, thermal blankets, immobilization and transport equipment, burn cures of varying severity, oxygen cylinders, a ventilator and a defibrillator. Diagnostic methods include glucometers, electrocardiogram, urine test strips, a rapid malaria test and an ultrasound machine. The intervention plan foresaw to find as most frequent medical problems burns, hypothermia, dehydration, traumatic injuries, aggressions and infectious pathology of diverse severity, as well as decompensations of untreated chronic pathologies (diabetes, hypertension, renal insufficiency, etc). All workers and volunteers of the NGO are offered vaccination, medical and psychological assistance, before and after the mission.. The rescue procedure from the initial assessment to the landing of refugees in the port assigned by the Italian navy is described in Fig. 1.

After being taken on board, each person undergoes an initial medical assessment. All sick patients, children and pregnant women are taken to the hospital area for more detailed management. Those classified as healthy receive further support in the waiting area; if lately they are reclassified as sick, they are taken as well to the hospital area. People usually stay in the boat 48–72 h until landing; those with a worrying condition are derived to Italian authorities to continue medical treatment. If during their stay on board the medical team considers that a patient needs urgent evacuation, is transferred to an Italian hospital by helicopter or speedboat, under the coordination of the Italian Maritime Rescue Coordination Centre (IMRCC).

Very few studies have been made about the conditions and challenges found in this kind of situation [7–9].

These studies show that, although boat migration represents only a fraction of illegal migration to Europe, it raises humanitarian and ethical issues for European and North African countries, as a non-negligible amount of the, end up in death tolls of shipwrecks in the Mediterranean Sea. Only one of them (7) is made specifically on board a medicalized ship. After rescuing 2656 people, they explain their protocol for action on board and suggest a method of medical assessment process that will allow for the effective discrimination of most serious cases.

Another study conducted in Italian reception centres with newly arrived refugees [10] showed that the most frequent conditions are those related to the migration experience and not representing a major threat for public health, as scabies, skin and respiratory infections. All these studies highlight that the misinformation in this situation is high, and more research involving the real burden of diseases is needed in order to identify the health needs of incoming migrants to arrange the appropriate response in terms of health services provision.

This paper aims to give an overview on characteristics and health conditions of rescued people by the NGO in the Central Mediterranean.

## Material and methods

A descriptive retrospective population study was conducted, including all refugees who were rescued from distress at sea by the NGO Open Arms from 1st July 2016 to 31st December 2018. There were no exclusion criteria.

Once people were rescued from the sea, and during the triage process performed by the medical personnel on board, the epidemiological data were recorded with the help of the rest of the ship’s personnel: sex, age and nationality.

Once the most severe patients had been treated and stabilized, the less severe symptomatic patients, as well as all pregnant women, were examined and a complete anamnesis was performed. In the event that it was considered necessary to evacuate a patient for medical reasons, the medical staff of Italian Maritime Rescue, responsible for the management of the operation, was contacted.

The data was recorded in each rescue in Excel Workbook in an anonymous way.

### Ethical aspects

The STROBE recommendations for the performance of observational studies have been applied [11].

The study was carried out in accordance with the Harmonized Tripartite Standards for Good Clinical Practice, following the current national regulations (Law 14/2007 pf Biomedical Research), and the Ethical principles derived from the Declaration on Helsinki. All data of the subjects participating in the study was anonymized. This research was conducted using clinical best practices. Need for informed consent was waived, as the study collected anonymized data. The study was approved by a review board within the sponsoring NGO.

### Variables examined

The study variables were:

-Demographic variables: gender, age, nationality; they were classified as minors if their reported age was less than 18. In the case of minors, if they were or not accompanied by family was registered; it was considered that they were accompanied if an adult recognized them as family, whatever the kinship.

-Clinical variables: pregnancy was registered in the case of women; most diagnoses were made based on signs and symptoms, and they were mostly clinical; diagnostic tests available on board were: glucose determination, rapid malaria test, electrocardiogram and an ultrasound device. In some cases, refugees were asked if they had suffered from physical or sexual violence. If cases of violence were detected, a full interrogation and physical examination was carried out, offering treatment for the urgent conditions detected in relation to it. The information was then handed over to the Italian health authorities for follow-up of the case. In case of a sexual assault reported in the previous 72 h, preventive treatment for a possible HIV transmission was offered. When a corpse was found, doctor on board gave a diagnosis of suspected cause of death. Corpses were washed and transported to the post of disembark, for later handling and identification.

### Data process and analysis

The data were described using measures of distribution, of central tendency and dispersion (mean and standard deviation). The statistical analysis was carried out using the SPSS 23.00® program.

Univariate analysis of our dataset included measures of distribution, central tendency (mean), and dispersion (standard deviation).

A multivariate analysis was not performed, due to the difficulty of linking the epidemiological and clinical data in some of the rescues, mainly those particularly extreme or with a very high number of people on board. In these situations, in the most serious or particularly important cases, the patient was recorded individually, but in some frequent conditions, as scabies, mild hypothermia, etc., the clinical information was not specifically referred to the epidemiological data.

## Results

### Sociodemographic data

**Fig. 2:**
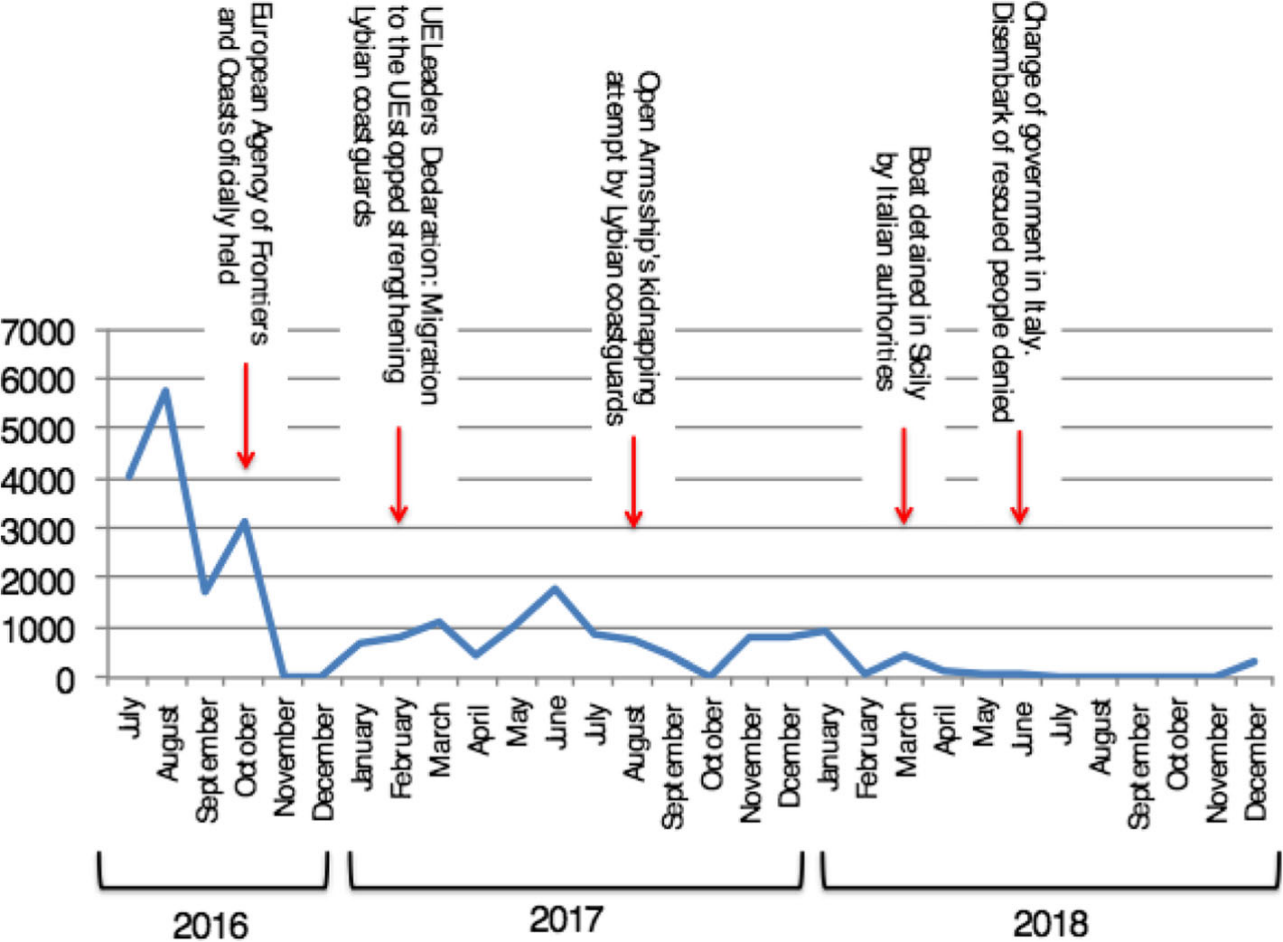
Open Arms monthly rescues in Central Mediterranean from July 2016 to December 2018. Some events related to the dynamics changes of the rescues are also shown

A total of 22.234 people were rescued from sea in this period of time. The number of rescued people according to months is showed in Fig. 2.

Demographic data were obtained from 9822 people; mean age among them was 19.9 years (SD 3.5); 2234 (22.7%) were minors, and 1368 (61.2%) of them non-accompanied; 1486 (15.1%) were women, 177 (11.9%) of them pregnant.

Between 1 and 1050 people could be rescued per refugee boat. Nationality, age structure and proportion of sick or injured people differed very importantly form one boat to another, depending on dates, city of departure, characteristics of the boat, circumstances of embarking and crossing, etc.

**Fig. 3:**
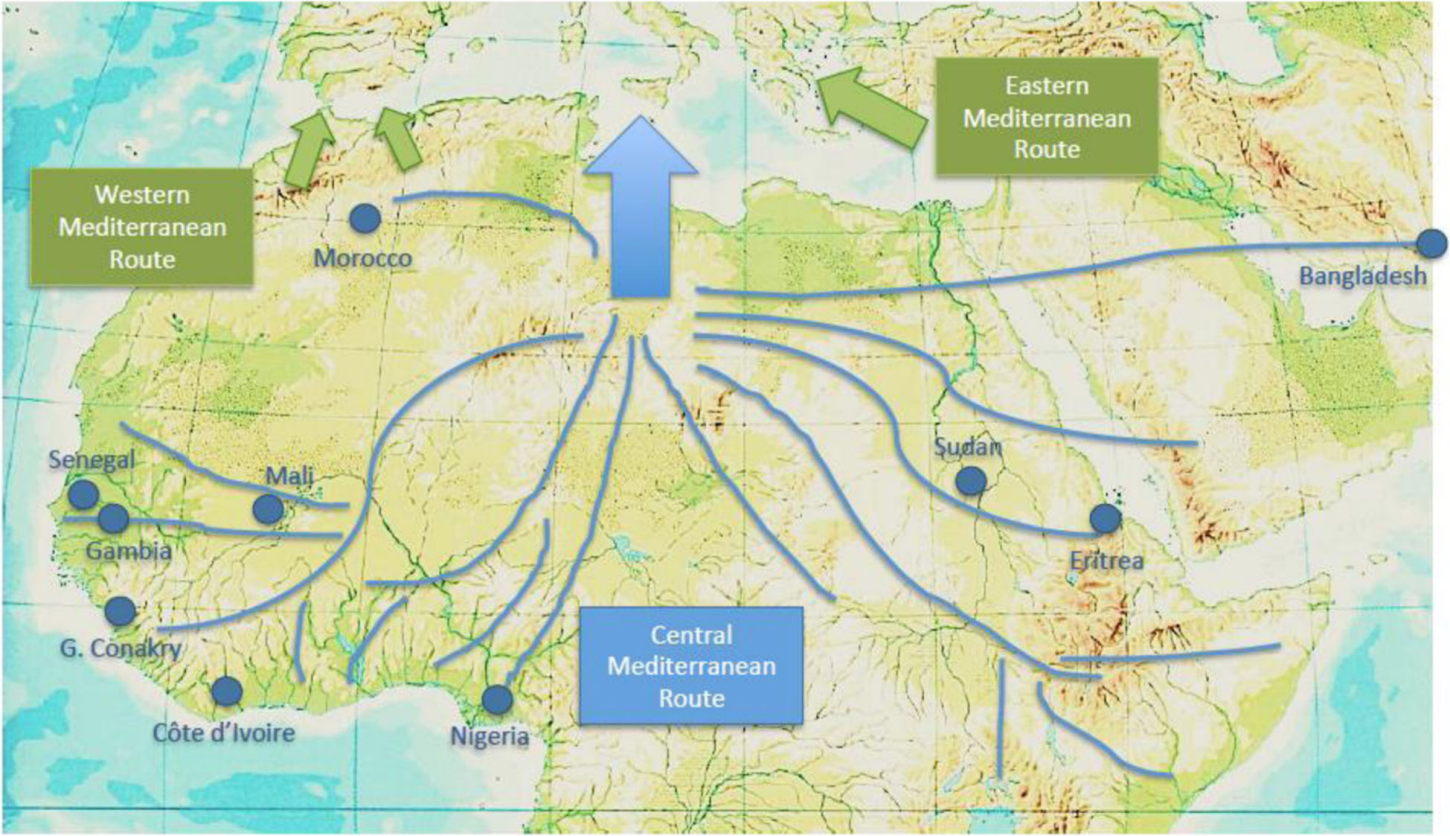
Ten main countries of origin of rescued people and most used routes to Lybia to the Central Mediterranean Route. Western and Eastern Mediterranean Routes are also shown (Source: data.unhcr.org/mediterranean)

Of all the rescued people, data on their nationality was obtained in 9383; among them, 7298 (74.3) were from Sub-Saharan Africa, 1444 (14.7) from Asia, and 1092 (11.1) from Northern Africa. All nationalities are specified in Table 1. Fig. 3 shows a map reflecting the main countries of origin and the most used routes to Lybia.

**Table 1.** Country of origin of rescued people by the NGO Open Arms in the Central Mediterranean from 1st July 2016 to 31st December 2018.

**Geographical area of origin**	**Cases (%)**
**Sub-Saharan Africa**	7298 (74.3)
Nigeria	1278 (13.1)
Eritrea	1215 (12.3)
Ivory Coast	830 (8.4)
Guinea Conakry	696 (7.2)
Mali	573 (5.8)
Sudán	458 (4.7)
Gambia	443 (4.6)
Senegal	441 (4.5)
Ghana	375 (3.9)
Somalia	218 (2.3)
Cameroon	215 (2.2)
Sierra Leone	68 (0.7)
Comoros Islands	67 (0.7)
Burkina Faso	62 (0.6)
Guinea Bissau	58 (0.6)
Liberia	50 (0.5)
Ethiopia	47 (0.5)
Equatorial Guinea	39 (0.4)
Togo	39 (0.4)
South Sudan	38 (0.4)
Democratic Republic of Congo	35 (0.3)
Chad	19 (0.2)
Níger	17 (0.2)
Benin	7 (0.06)
Central African Republic	6 (0.06)
Mauritania	3 (0.03)
Namibia	1 (0.01)
**Asia**	1444 (14.7)
Bangladesh	981 (9.9)
Pakistan	274 (2.8)
Syria	86 (0.9)
India	44 (0.5)
Palestina	23 (0.2)
Yemen	15 (0.1)
Nepal	14 (0.1)
Sri Lanka	5 (0.05)
Afghanistan	1 (0.01)
Tibet	1 (0.01)
**North Africa**	1092 (11.1)
Morocco	750 (7.6)
Libya	162 (1.7)
Egypt	117 (1.12
Argelia	53 (0.6)
Tunisia	10 (0.1)

### Clinical data

Among the 22,234 people rescued, 4516 (20.3%) reported symptoms.

Scabies was the most frequent pathology, being suspected in 1817 (8.2%) people. Other infectious diseases were diagnosed in 91 (0.4%). Thirty-five (0.16%) patients suffered some complication from their chronic diseases. Acute injuries due to trauma, burns, aggressions, and bullet or bladed weapon wound were reported in 135 (0.6%) cases. All the diagnosed pathology is summarized in Table 2.

**Table 2.** Analysis of conditions and injures found among the refugees from the total number of patients: 4516/22234 (20.3%).

**Symptoms/Suspected diagnoses**	**Number (%)**
**Infectious diseases**	1899 (42%)
Scabies	1817
Respiratory infection	12
Suspected infectious diarrhea	13
Pyelonephritis	1
Suspected TB	8
Conjunctivitis	5
Otitis	2
Cutaneous infections	19
Odontogenic infection	9
Septic shock	1
Acute febril illness	21
**Hypothermia**	262 (5.8%)
**Heat stroke**	1 (0.02%)
**Drowning**	1 (0.02%)
**Dehydration**	61 (1.3%)
**Complication from chronic diseases**	33 (0.7%)
Renal disease	3
Schizophrenia	3
Heart disease	5
Deaf	2
Diabetic ketoacidosis	5
Asthma	15
**Acute injuries**	135 (3%)
Trauma	28
Bullet wound	6
Bladed weapon wound	36
Chemical burns	58
Other burns	4
Bites	2
Lower limb amputation	1
**Gynecological problems**	15 (0.3%)
Suspected abortion	2
Recent birth	2
Endometritis	2
Umbilical cord infection	1
Labor contractions	4
Birth on board	1
Gravidic hyperemesis	1
Vaginal ulcer	2
**Other conditions**	2100 (46.5%)
Sea sickness	1994
Posttraumatic stress	45
Hernias	6
Hematemesis	6
Severe malnutrition	4
Hypoglycemic coma	20
Gasoline vapors inhalation	3
Other	22

Both healthy and sick refugees were transferred to Italian authorities on disembark, respectively to an Italian homeland or to a treatment facility; 24 people had to be evacuated prematurely during the boat trip; 17 of them were made from the high seas: 4 septic people, 3 due to heat stroke, 3 premature neonates, 3 women in labor, 2 infants with malnutrition and severe dehydration, 1 injured by firearm, and 1 infant with hydrocephalus. The other seven were evacuated when they reached land: 3 severe chemical burns, one spleen hematoma, one endometritis, an infant with cord infection and a man with severe malnutrition.

One woman gave birth 10 minutes after being rescued; the baby had to be reanimated during 10 minutes, being in good condition afterwards; they were both urgently transferred to an Italian hospital.

To note, 100% of interviewed people claim to have suffered or witnessed physical violence and 100% of women to have been victims of sexual violence.

Three people died on board: The first one was a 29 year-old patient from Ghana, being the probable cause of death a heat stroke though differential diagnose was made with bacterial meningitis; high-spectrum antibiotic and thermal measures were taken and sedation and oro-traqueal intubation were performed; hospital transfer was delayed due to the possible infectious risk and finally the patient died at the hospital; although the diagnosis of meningitis was not confirmed, all personnel involved in the treatment of the patient as well as his close contacts were treated with ciprofloxacin 500 mg single dose as a preventive measure. The second one was a three-month-old baby of Eritrean nationality, due to a generalized sepsis, while waiting to be evacuated and transferred to a hospital. On the boat, antipyretic treatment and broad-spectrum antibiotic were administered, and the third 1 a 21-year-old man from Eritrea, who suffered a cardiac arrest, witnessed during the rescue; advanced cardiac resuscitation was performed for 30 min, but he could not be resuscitated. The suspected cause of death was a hydroelectrolitic imbalance due to extreme dehydration and starvation.

In addition to all the people rescued alive, 74 corpses were recovered,, 58 of them in rubber boats, 13 in wooden boats and 3 floating in the water. All of them were recognized by the medical staff and transferred to port for identification and burial. The probable causes of death were recorded, being these drowning, dehydration or inhalation of gasoline vapors. An indeterminate number of missing people were not found.

## Discussion

Here we describe socio-demographic and clinical conditions on board the rescue boats of the NGO Open Arms.

Because of the inner characteristics of the rescue operations, which are usually massive rescues that include hundreds of people, in which the action must be quick in order not to create a delay that impairs the attention of further people, data are scarce and in some cases not collected in depth. This was particularly notorious during the first missions carried out in 2016. At that time the rescue boat was Astral; its small size did not allow the transfer of the rescued people to ports located many miles away, so they were transferred to other ships present in the area soon after the rescue.

Some clinical conditions, such as mild dehydration or hypothermia, and some symptoms such as unidentified pain or discomfort, as well as anxiety attacks and other mild mental disorders were not recorded, although they are probably very frequent in this context [12, 13]; in many cases the communication was severely hampered due to language barrier, in need of an interpreter, usually another refugee with knowledge of several languages, in some cases only non-verbal communication could be carried out.

Also, the initial diagnoses are mostly clinical, since the only diagnostic tests on board are glucose determination, rapid malaria test and a portable ultrasound; therefore many diagnoses should be interpreted as “provisional” data, meaning that the diagnoses are suspected and with a focus on those needing urgent treatment. A multivariate analysis could not be performed to identify factors, as age or country of origin, associated with the development of some diseases, due to the difficulty of linking the epidemiological and clinical data in some of the most extreme rescues.

Despite these limitations, we believe that these are very valuable data, since there are very few reports on the characteristics of refugees during their migration [6, 14, 15]; as in every context, epidemiological data are essential to plan appropriate interventions and to optimize resources [16]. It is also an issue of exceptional gravity and topicality due to the number of people involved, the very harsh living conditions and the high number of deaths during the migration [17]; according to the United Nations High Commissioner for Refugees (UNHCR), this is the biggest migratory and humanitarian crisis in Europe since World War II [18]. This study has a number of policy and practice implications, as it shows the failure of organizing alternatives for people to migrate and seek asylum in an organized manner, pushing people to a most dangerous migration and into the hands of smugglers [19]. Since the study was completed, the situation of civil society organizations working in the Central Mediterranean has worsened in a very significant way; the obstacles and difficulties imposed by European governments have prevented the departure of rescue ships since January 2019, making this route the most deadly migratory route nowadays [6].

The average age of the rescued persons is approximately 20 and almost 23% were minors, the majority of them unaccompanied. These data are similar to data reported by the European Union on the demographic characteristics of asylum seekers [20]. This population is extremely vulnerable both to violence, abuse and traumatic stress reactions [21, 22]. Other major concerns about children’s health in this context are no or incomplete immunization, undiagnosed congenital disorders or poor nutrition that could lead to hampered development [23].

Another particularly vulnerable population are women, who represented 15% of all rescued people in our study; this figure is lower than the general percentage of women seeking asylum in the European Union, which corresponds to approximately one third of the total [8]. This difference may be due to the fact that this route is particularly tough, including crossing several countries before reaching the detention centers in Libya. In our study, 100% of interviewed women reported to have suffered sexual violence during migration, particularly during their stay in Libya. Almost 12% of women were pregnant, most of them travelling alone; it is estimated than in a disaster situation, 4% of population is made up of pregnant women, of which 20–30% will experience an unpredictable obstetric complication or surgical intervention [24]. The absence of timely antenatal care puts mothers and babies on a higher risk of complications [25, 26]. In this case, none of the women had access to antenatal care during pregnancy; an obstetric ultrasound was performed on board when possible, and pregnancy was reported to the authorities at disembark, with the aim of continuing care.

The majority of rescued people came from Sub-Saharan Africa (74.3%), being the most frequent countries of origin Eritrea (12.3%), Nigeria (13.1%), Ivory Coast (8.4%) and Guinea Conakry (7.2%). Though the three major countries of asylum seekers in last years in the European Union are Syria, Afghanistan and Iraq, Nigeria, Ivory Coast and Guinea Conakry are among the ones with largest relative increases compared to previous years (18). Syria, what was the origin of the vast majority of refugees rescued in the previous mission of the NGO in Lesbos Island, was in this case the country of origin of 462 (2.1%) people; there were refugees from as far afield as Sri Lanka, Bangladesh, Nepal and Comoros Islands.

Seventy-four corpses were recovered during the rescue operations, most of them in rubber boats, also known as dinghies. Both thus kind of boats and the wooden ones, which are also used, are very fragile boats, which sink quickly in case of any crack in their structure. Gasoline burns are more frequent in rubber boats, since the bottom of the boat is easily filled with water and gasoline, while asphyxia from motor smoke is more frequent in wooden boats, due to the agglomeration of people at various height levels. The total number of deaths is unknown and probably much higher, since in several occasions half-sunken rafts were found with only a few corpses and in others the refugees refer people dead during the crossing, and thrown into the sea.

Main diagnoses on board were directly related to the precarious living conditions through migratory route, violence and complications of chronic diseases due to lack of care. The most frequent diagnoses were scabies, fever, respiratory and digestive tract infections, mostly as a direct result of poor living conditions in transit. This is similar to other reports by other NGOs that have worked in the area and summarized by WHO [12–14, 21].

On the whole, 42% of diagnoses were communicable diseases. Emphasis is often placed on these [27]; nevertheless we must not forget that they are primarily diseases of poverty and their predominant role indicates that basic health needs are not covered. Disease prevention and control efforts should be taken in front of these diseases; this is particularly difficult in overcrowded situations. In the case of particularly serious diseases such as tuberculosis, the effort must be greater; in our case the suspected patients remained in a separate and well-ventilated area. No contagion was reported among the personnel on board.

Violent trauma, injuries and burns were present in almost 3% of all attended patients, with a wide variety of causes: accidental trauma due to lesions during journey, wounds due to deliberate violence and burns; a special type of chemical burns found in this situation was gasoline wounds, due to the mixture of salt water with fuel that is often spilled inside the boats and stays attached to the clothing and body, causing deep burns due to prolonged skin contact. Recognition and rapid management of this situation is fundamental and the first action must be to withdraw the contact with the toxic, removing the clothes, then the wound should be cleaned and covered with a universal chelator [28].

Non-communicable diseases, including complications of chronic diseases were found in a low percentage in this population, in contrast to other reports, which account up to 40% of all diagnoses [12, 21]. This may be due to the low mean age of our group; there is also the possibility that in a transit population, in which people prioritize basic needs, those with mild symptoms on board may not recount. In addition, the hardness of the migratory process in general, an particularly of this route, makes that the people who carry it out are generally healthy [29]. Management of these conditions in a mobile context is highly challenging, as discontinuation of chronic controls and medications may cause exacerbations of previously well controlled non communicable diseases [30], which globally account for 63% of deaths, including 14 million people of premature deaths [31]. Other conditions related specifically to the maritime route and meteorological conditions, as drowning, hypothermia or heat stroke, were also reported.

The proportion of mental health disorders reported was very low in our series compared with other studies [32]. This is probably due to underreporting previously commented, as well as the inner characteristics of the rescues, in which the refugees are still in a very vulnerable transit situation, and the staff is mainly dedicated to the emergency situations. In addition, the overcrowding on board generally did not permit the needed privacy to attend these issues. Depression, anxiety, sleep disturbance and post-traumatic stress disorder are as common as 20% in situations of forced migrations and abuse according to WHO [33]. One study performed in Greece found that 50% of refugees presenting for mental health screening were diagnosed with mental health conditions and, of these, 60% had experienced potentially traumatic events in their country of origin and 89% during migration [34]. In our study, 100% of interviewed people reported that kind of event, including sexual abuse, violence against themselves or others, or having been witnesses of murders.

In relation to the data presented in this article, the NGO Open Arms continues its work in the Central Mediterranean, prioritizing the rescue and treatment of migrants. Greater emphasis should be placed on mental health management; trauma-focused therapies have been shown to be effective within refugee populations [35], including in acute rescue situations. In addition, efforts have been made to denounce current migration policies, which make it difficult to rescue people in extremely vulnerable situations and in imminent danger of death. Regarding to it, better coordination between the rescue boats and the authorities would make it possible to ensure that patients are followed up. As this is a particularly fragile situation changing constantly, an integrated information system with the possibility of monitoring the state of health of migrants upon their arrival in the Mediterranean, and sharing information among the different stakeholders regarding the burden of illness and the profile of migrants is essential to elaborate a coordinated response. Studies done in the field that objectively analyze the reality of the current situation are much needed.

## Conclusions

This study shows the work of the NGO Open Arms during its rescues of refugees in the Mediterranean Sea.

The large number of people rescued highlights the catastrophic effect on migrants’ health of European policies, which seek to outsource borders, overlapping the desire to restrict migratory movements on the humanitarian and welfare aspects of migrants. The chosen route through the Central Mediterranean has become one of the most dangerous in the world; it includes serious violations of human rights, leading to the vulnerability and extreme marginalization of migrants.

This is a matter of global health significance, since it implies to place equal value in human life above all other considerations. An integrated information system and a coordinated response are basic to improve the situation in the area.
